# The Suitability of FGF21 and FGF23 as New Biomarkers in Endometrial Cancer Patients

**DOI:** 10.3390/diagnostics10060414

**Published:** 2020-06-18

**Authors:** Aneta Cymbaluk-Płoska, Paula Gargulińska, Anita Chudecka-Głaz, Sebastian Kwiatkowski, Ewa Pius-Sadowska, Bogusław Machaliński

**Affiliations:** 1 Department of Gynecological Surgery and Gynecological Oncology of Adults and Adolescents, Pomeranian Medical University, Al. Powstańców Wielkopolskich 72, 70-111 Szczecin, Poland; p.gargulinska@wp.pl (P.G.); anitagl@poczta.onet.pl (A.C.-G.); 2 Department of Obstetrics and Gynecology, Pomeranian Medical University, Al. Powstańców Wielkopolskich 72, 70-111 Szczecin, Poland; kwiatkowskiseba@gmail.com; 3 General Pathology Department, Pomeranian Medical University, Al. Powstańców Wielkopolskich 72, 70-111 Szczecin, Poland; ewapius@wp.pl (E.P.-S.); machalin@pum.edu.pl (B.M.)

**Keywords:** FGF21, FGF23, leptin, endometrial cancer, obesity

## Abstract

Endometrial cancer is one of the most common cancers of the reproductive organ in women. The incidence of it increases from year to year. In our study we assessed role of FGF21 and FGF23 in the diagnostics of patients with endometrial cancer. The study involved 182 patients, who were undergoing abrasion due to perimenopausal bleeding. FGF21, FGF23, and leptin concentration were quantified in serum by multiplex fluorescent bead-based immunoassays (Luminex Corporation). The median of FGF21 protein (181.8 pg/mL) as well as leptin (16.9 ng/mL) in patients with endometrial cancer was statistically significant higher compared to median of those proteins among patients from control group (152.1 pg/mL and 14.1 ng/mL, respectively). However, no significant differences were found in these groups at median FGF23 concentrations. For FGF21 and leptin, the AUC values were 0.81/0.79, while FGF23, the AUC values was 0.66 for all study patients. Leptin and FGF21 concentrations were statistically significantly higher in patients with poorly differentiated G3 tumors compared to patients with moderately differentiated G2 tumors and with moderately differentiated G2 with highly differentiated G1 respectively: *p* = 0.02/*p* = 0.03 and *p* = 0.02/*p* = 0.005. FGF21 appears to be useful as a diagnostic as well as prognostic factor in patients with endometrioid endometrial carcinoma.

## 1. Introduction

Endometrial cancer is one of the several malignancies of the genital organs characterized by incidence rates that in recent years have been increasing rather than decreasing. Trends of incidence rates in endometrial cancer age-standardized increased by 1757 in Central Europe in 1990–2017, which makes Central Europe the fourth place around the world, with a mortality rate that decreased by 0.447 during this period [1]. Most patients receive their diagnosis at an early stage of the disease. Five-year survival in patients with stage I cancer is about 84%, and in this group, disease recurrence occurs only in 15% of cases. In more advanced stages recurrence rate ranges from 35–60%. As many as 80% of endometrial cancer cases are associated with obesity, insulin resistance, and type II diabetes mellitus [2,3]. Mechanisms responsible for the development of insulin resistance are quite well understood. In 1997, Klotho discovered a gene initially considered to be an anti-aging gene. Currently, the protein transcripts of the Klotho gene are being studied for their involvement in carcinogenesis. Studies in breast cancer cell lines have revealed that the Klotho gene is an inhibitor of the IGF-1 pathway and an activator of the FGF pathway [4,5]. Taking into account the endometrial cancer risk factors as well as the reports on oncogenic effects of Klotho transcripts possibly exerted via fibroblast growth factor receptors, we decided to carry out a detailed study of the FGF subfamily responsible for systemic metabolic processes [6].

Fibroblast growth factors (FGFs) and their receptors (FGFRs) play a complex role in the control of numerous systemic processes. They control the tissue healing process and angiogenesis and are thus an important element responsible for the maintenance of homeostasis. Anomalies in the FGF/FGFR axis signaling may lead to disturbances in these processes and thus result in pathological conditions such as malignancies [7,8].

Fibroblast growth factor 23 (FGF23) is a member of an FGF subfamily of hormone-like FGFs, which also encompasses FGF19 and FGF21 [9]. FGF23 is produced mainly within osteoblasts, but its expression is also observed in other tissues such as the stomach, liver, muscles, or the mammary gland. The target organs for FGF23 are the kidneys. Physiologically, the Klotho protein, being formed predominantly within the kidneys, is a cofactor required for proper activity of FGF23. To date, no direct relationship has been identified between the overexpression of FGF23 and the pathogenesis of endometrial cancer. However, reports have been published on a correlation between increased leptin levels and increased serum concentrations of FGF23. Considering the fact that increased body mass, dyslipidemia, and increased leptin levels are factors involved in the pathogenesis of endometrial cancer, one should expect that patients with endometrial cancer should also present with higher levels of FGF23 [10,11,12].

Fibroblast growth factor 21 (FGF21) is another member of the family of FGFs with endocrine activity. The highest expression of this factor is observed in hepatic and pancreatic cells; less expression is observed in adipocytes, myocytes, and duodenal cells. In a manner similar to FGF23, FGF21 requires the Klotho protein as a cofactor. FGF21 exerts its effects on the target tissue to regulate the metabolism of glucose and lipids [13]. It also has a beneficial impact on bodyweight reduction. In addition, FGF21 affects cell proliferation and migration via the PI3K/AKT signaling pathway [14]. The role of FGF21 in carcinogenesis has not yet been fully explained. Reports were published on overexpression of FGF21 in breast cancer, potentially making the compound a highly sensitive biomarker of this disease. Considering the fact that obesity, metabolic disorders, and estrogen imbalance are important factors in the pathogenesis of both breast and endometrial cancer, the potential involvement of FGF21 is also pointed out in endometrial cancer patients [15].

Leptin is a protein composed of 146 amino acids, coded by the obese (ob) gene. It is a hormone produced mainly in adipose tissue and plays an important role in the control of metabolic processes and the energetic balance. It has been proven that by affecting cell proliferation, it participates in the growth of cancers, including endometrial cancer. Its carcinogenic effect is caused by the activation of JAK/STAT 16 signal pathways [16]. Leptin increases the expression of FGF21 secreted in fat tissue and FGF 23 in osteoblasts.

### Objectives

To assess the role of FGF21 and FGF23 in patients with endometrial cancer diagnostics, determining whether their concentration correlates with the degree of clinical stage and histopathological differentiation in endometrial cancer patients.

## 2. Material and Methods

The study involved 182 patients who were undergoing abrasion due to perimenopausal bleeding. Excluded from the study were patients with a history of treatment for other oncology diseases, liver dysfunction, inflammation process, or incomplete data. In addition, patients who had renal insufficiency were excluded from the study due to elevated FGF levels. In this case, it would be necessary to determine a correction factor depending on GFR and creatinine, which was not done [17]. All the patients who were involved in the research signed informed consent and agreed to all the requirements. The study protocol was approved by the Ethical Committee of the Pomeranian Medical University (Resolution number: KB-0012/77/12 of the Bioethics Committee of the Pomeranian University of Medicine in Szczecin on 13 October 2012).

All patients qualified for the study were measured and weighed. These results were used to calculate BMI.

The formula below was used to calculate BMI:

$$BMI=\frac{weight\left[kg\right]}{height{\left[m\right]}^{2}}$$

After calculating the BMI index, it turned out that the lowest BMI of the patients included in the study was 22. It was decided to divide them by their respective BMI into three groups:

- 22 < BMI < 25, *n* = 36
- BMI 25–30, *n* = 77
- BMI > 30, *n* = 69

All patients had waist circumference (WC) measured and were divided on this basis into two groups:

- WC < 100 cm, *n* = 42
- WC > 100 cm, *n* = 140

Patients’ blood glucose levels were determined. The measurement was taken on an empty stomach, from venous plasma. The correct result was 72–99 mg/dL (4.0–5.5 mmol/L). In the patients whose results exceeded the higher value, we found glucose intolerance.

- Fasting glucose intolerance, No, *n* = 161
- Fasting glucose intolerance, Yes, *n* = 21

All patients were asked about medical conditions and medication. Due to the effect on the tested protein on glucose metabolism, it was decided to divide the patient into a group depending on the occurrence of DM type2:

- DM type 2, yes, *n* = 101
- DM type 2, no, *n* = 81

Detailed patient characteristics relating to the endometrial cancer risk factors are shown in Table 1.

Patients qualified for the study underwent the following procedures: abrasion, hysteroscopy, or radical surgery. Radical operation means total hysterectomy and bilateral salpingo-oophorectomy with lymph nodes concerned only the study group, i.e., patients with histopathologically confirmed diagnosis of endometrial cancer after abrasion or hysteroscopy. The control group included patients after abrasions or histrescopias with histopathologically confirmed endometrial polyps or with normal endometrium. Patients with atypical and simple hyperplasia and patients receiving gestagens were excluded from the study.

Division of patients into groups depending on the histopathological diagnosis:

- Endometrial cancer patient, *n* = 98
- Patients with normal endometrium, *n* = 51
- Patients with endometrial polyps, *n* = 33

With the above histopathological results, 2 groups and 2 subgroups were created:

- Group A—Endometrial cancer patient, *n* = 98,
- Group B—Patients with benign endometrium changes, *n* = 84
- Subgroups B1—Patients with normal endometrium, *n* = 51
- Subgroups B2—Patients with endometrial polyps, *n* = 33

In group A 82, patients were diagnosed with endometrial endometrioid carcinoma and 16 patients with non-endometrial endometrioid carcinoma.

During routine preoperative examinations, an additional 5 mL of blood was collected from each of the patients who consented to the examination. The blood was centrifuged, and obtained serum was stored in a freezer at −70 °C.

Multiplex fluorescent bead-based immunoassays (Luminex Corporation, Austin, TX, USA) and commercial Bio-Plex Pro RBM Human Metabolic Panel 2 (Bio-Rad, Hercules, CA, USA) were used to measure leptin, FGF21 and FGF 23 concentrations in serum. The tests were performed by adding 30 μL of standard, control, and action on the plate, after adding 10 µL blocker to all wells of the plate together with 10 µL downstream of the antibody capture multiplex. The plate was incubated for one hour at room temperature with shaking. After this step, the wells were washed individually three times using the test buffer. 40 μL of antibody detection cocktail was added with a pipette to each well. The plate was then placed tightly closed on a shaker and incubated for 1 h at room temperature. After the shaking phase, a streptavidin-phycoerythrin mixture was added to the plate, thereby diluting it. Then, it was again incubated with stirring for 30 min in the dark at room temperature. After washing the microspheres in each well, the assay buffer was added again and shaken for 30 s at room temperature. The plate was then read and analyzed on a Luminex analyzer.

The Statistica 10 PL software was used for statistical calculations. For the basic descriptive analysis characterizing the examined group of patients—min, max, range, mean and median values were used. The distribution of data in the studied group of patients did not meet the criteria for using parametric tests, because it was not normal and homogeneous, therefore non-parametric tests were used. Mann-Whitney’s U-test was used for the comparison between the two groups, while Kruskal–Wallis test and Dunn’s post-hoc test were used to compare three groups. Because of the non-parametricity of the traits, for the analysis, the Spearman’s rank correlation coefficient was used. The receiver operating characteristic (ROC) was used as a classification tool to assess the combined sensitivity and specificity of the parameters tested. Multivariant logistic regression, which retained case matching and control, was used to estimate the ORs and the 95% CIs for the associations between the risk factors and protein studied and the endometrial cancer risk. A *p*-value <0.05 was considered indicative of statistical significance.

## 3. Results

There were no statistically significant differences between the median age of the examined and control patients (54.36 vs. 53.22 years) (Table 2). In the group of patients with endometrial cancer, an increased median BMI than in patients of the control group was found, but the difference was not statistically significant (29.81 vs. 26.99 kg/m^2^). Statistically higher percentages of patients diagnosed with type II diabetes mellitus, as well as patients with fasting glucose intolerance were observed in patients with endometrial cancers as compared to the control group (*p* = 0.02 and 0.03, respectively). The frequency of both DMT2 and fasting glucose intolerance was significantly higher in patients with BMI > 30 kg/m^2^ compared to non-obese patients. No statistically significant differences were observed in the analyses of WC values in patients with endometrial cancer and the control group.

### 3.1. Study Group Protein Analysis

Significantly higher median leptin, FGF21, and FGF23 concentrations were noted in obese patients compared to median concentrations observed in patients with normal weight. We found a significantly higher median concentration of leptin and FGF 21 in patients from group A with diabetes and obesity, compared to patients from group B with the same metabolic diseases (Table 3).

**Figure 1:**
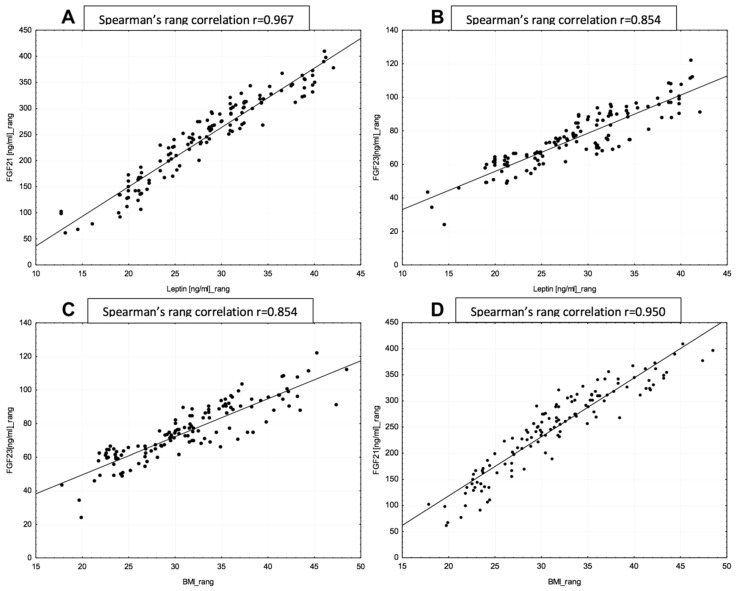
(**A**) Correlation between leptin and FGF 21 level. (**B**) Correlation between leptin and FGF 23 level. (**C**) Correlation between FGF23 level and BMI. (**D**) Correlation between FGF21 and BMI.

Median of leptin and FGF21, but not of FGF23, were statistically higher in patients with DMT2 and fasting glucose intolerance. Statistically significant correlations were also observed between WC values and the levels of leptin and FGF21 with corresponding coefficients amounting to r = 0.87 (*p* = 0.02) and r = 0.81 (*p* = 0.001) respectively. Strong correlation was also observed between leptin and FGF21 levels as well as between leptin and FGF23 levels, corresponding coefficients being r = 0.97 (*p* = 0.003) and r = 0.85 (*p* = 0.01), as shown in Figure 1. No statistically significant differences were observed with respect to median levels of all proteins of interest, considering the hormonal status of patients.

The median concentration of FGF21 protein (181.8 pg/mL) and also leptin (16.9 ng/mL) in the study group was significantly higher compared to median concentrations of those proteins among patients from control group 152.1 pg/mL and 14.1 ng/mL, respectively), as shown in Table 4.

**Figure 2:**
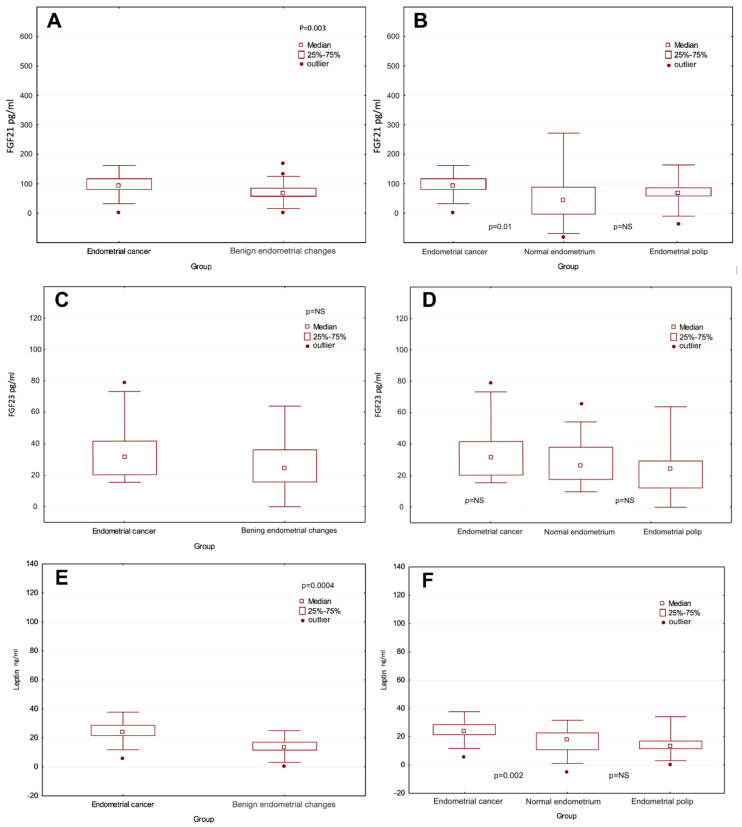
(**A**) FGF21 level distribution in the study and control group. (**B**) FGF21 level distribution in the subgroups. (**C**) FGF23 level distribution in the study and control group. (**D**) FGF23 level distribution in the subgroups. (**E**) Leptin level distribution in the study and control group. (**F**) Leptin level distribution in the subgroups.

We also found differences in the median concentrations of both FGF21 and leptin in patients with endometrial cancer compared to those in the median concentration in the group of patients with endometrial polyps. They were statistically significantly higher in the examined group of patients (16.9 ng/mL; 15.3 ng/mL, and 181.8; 140.7 respectively), *p* = 0.01/*p* = 0.004. However, no significant differences were found in these subgroups at mean FGF23 concentrations (Figure 2). There were no statistically significant differences between the median concentrations of FGF21, FGF23, and leptin in the subgroup of patients with normal endometrium and the median level of this protein among patients with endometrial polyp. Data on the median concentration of the tested proteins in individual groups and subgroups are presented in Table 5.

### 3.2. Comparative Analysis Based on the Presence of Risk Factors

Considering the currently negative prognostic factors of endometrial cancer, statistically significant differences between endometrial vs. non-endometrial types have been demonstrated only in relation to FGF-21 protein. Serum concentrations of leptin and FGF21showed differences depending on lymph node occupation *p* = 0.02. Median leptin levels and FGF21 were significantly higher among patients with lymph vessels occupation, respectively (*p* = 0.003/*p* = 0.01) compared to the control group. The study showed for all tested proteins: leptin, FGF21, and FGF23 statistically significant differences between subgroups in patients with G3 versus well-differentiated G1. In addition, median leptin and FGF21 concentration were significantly higher in patients with poorly differentiated G3 tumors compared to patients with moderately differentiated G2 tumors and moderately differentiated G2 with well-differentiated G1, respectively: *p* = 0.02/*p* = 0.03 and *p* = 0.02/*p* = 0.005. We observed no statistically significant differences in FGF23 median between poorly and well-differentiated cancer cells.

Significantly higher values of median leptin and FGF21 were also demonstrated in cases of deep myometrium infiltration (*p* = 0.04, *p* = 0.002, respectively). We observed significantly higher median of all proteins, i.e., leptin, FGF21, and FGF23 in patients with more advanced disease (FIGO III/IV) as compared to patients with less advanced disease (FIGO I/II).

### 3.3. ROC Curve Analysis and Test Sensitivity/Specificity Evaluation

To check the diagnostic capabilities of leptin, FGF21, and FGF23, ROC curves were plotted, and areas under ROC curves (AUC) were calculated.

**Figure 3:**
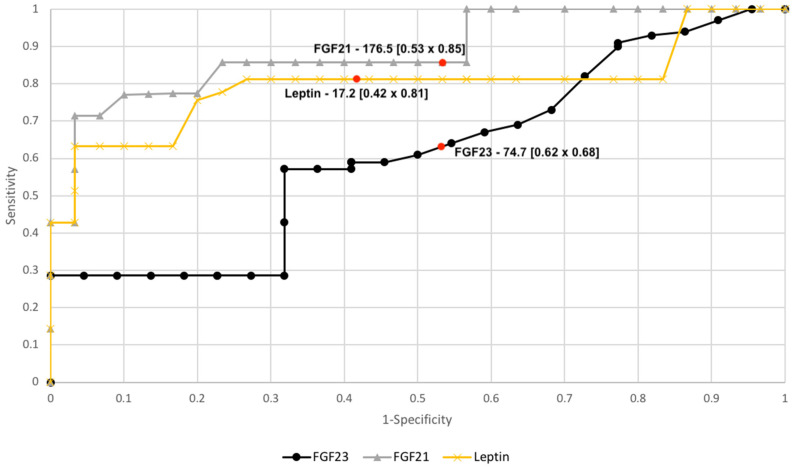
The receiver operating characteristic curves for proteins in endometrial cancer and benign endometrial changes.

**Figure 4:**
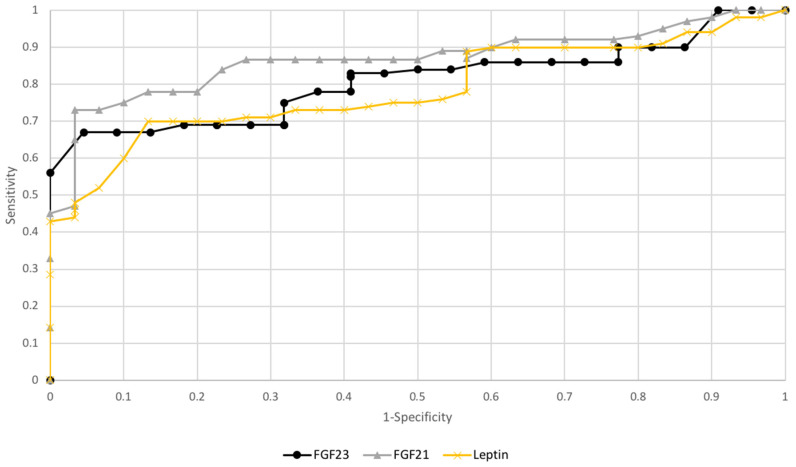
The receiver operating characteristic curves for proteins in endometrial cancer depending on hormonal status.

FGF21 and leptinAUC values were 0.81/0.79, while FGF23, the AUC values were 0.66 for all study patients. AUC value for FGF23 in subgroups premenopausal patient was 0.82 and postmenopausal 0.78 (see Figure 3 and Figure 4).

**Figure 5:**
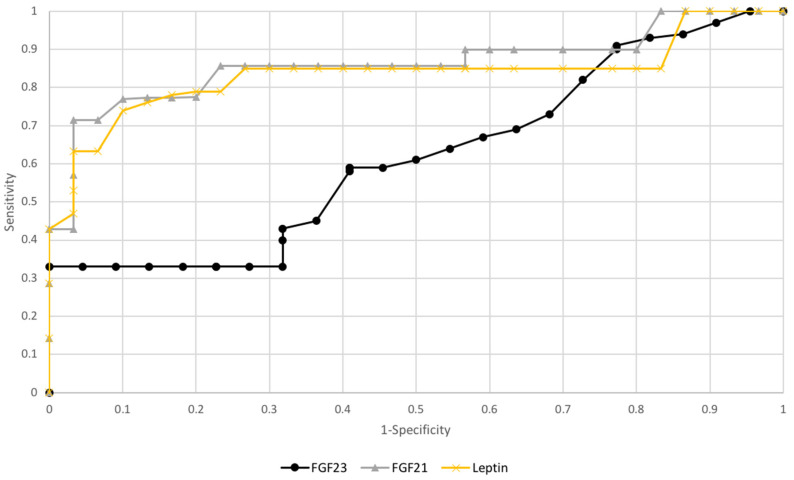
The receiver operating characteristic curves for proteins depending on the stage of endometrial cancer.

**Figure 6:**
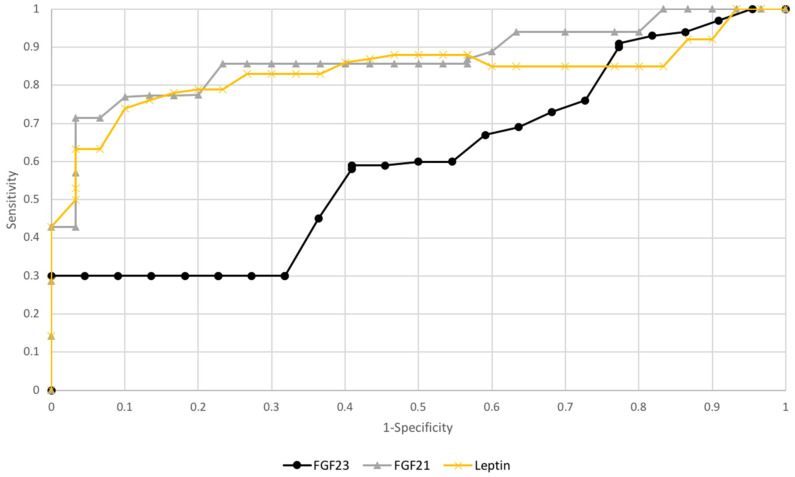
The receiver operating characteristic curves for proteins depending on the grading of endometrial cancer.

Checking the diagnostic possibilities of leptin and FGF21 in case of differentiation between high (FIGO III and IV) and low (FIGO I and II) stages of clinical advancement of the tumor and forecasting the degree of histopathological differentiation of the tumor (G1 vs. G3) based on the AUC curve was as follows: 0.79/0.78 and 0.80/0.82 (see Figure 5 and Figure 6). The AUC of the ROC curve for the FGF23 protein relating to differentiation in the clinical stage of the tumor and histopathological differentiation of the tumor are 0.54 and 0.62, respectively. Definitely higher sensitivity than specificity for leptin protein was found in all patients (84%/72%). The worst sensitivity and specificity were noted for FGF23 (60%/52%) compared to FGF21 (86%/77%), as shown in Table 6.

Having applied multivariate logistic regression analysis for the risk of the development of endometrial cancer, in the final model, independent risk factors were found: BMI, FGF21, and leptin level. Classification determined on the basis of the cut-off values for leptin (17.2 ng/mL) gives OR 1.24. OR 1.21 was for cut-off (176.5 pg/mL) values for FGF21 (Table 7).

## 4. Discussion

Fibroblast growth factors are a large group of proteins with varied functions. The sixth subfamily of FGFs consists of three endocrine factors, FGF19, FGF21, and FGF23. Fibroblast growth factor 21 (FGF21) is a strong metabolism regulator. It regulates the storage and use of carbohydrates. FGF21 is present mainly in the liver, white adipose tissue, skeletal muscles, and the pancreas [9].

FGF23 is released from osteoblasts and osteoclasts. It regulates the metabolism of vitamin D3 and phosphates to inhibit the reabsorption of phosphates in renal tubules. FGF23 is the key humoral regulator of phosphate homeostasis [17,18]. FGF23 induces the synthesis of proinflammatory factors TNFα and macrophages within splenocytes as well as IL6 and CRP in hepatocytes. This may play an important role in the pathogenesis of generalized inflammations. Considering the fact that metabolic syndrome is the key risk factor for endometrial cancer, the examination of serum levels of FGF21 and FGF23 in endometrial cancer patients appears warranted. In our study, besides determinations of the aforementioned FGF family members, we also examined serum leptin levels in patients with endometrial cancer, endometrial polyps, and healthy endometrium [19].

Fat tissue is currently seen as an endocrine adductor of adipocytokines. The most known of adipocytokines is leptin, which plays a key role as a marker of obesity. Chronic inflammation that accompanies obese people and people with metabolic disorders coexists with hyperinsulinemia and hyperleptinemia. These hormones appear to have a key role in the proliferation of endometrial cancer cells in obese individuals [20]. Many studies have shown higher levels of leptin in patients with endometrial cancer [21]. In addition, our current study has confirmed earlier reports that higher levels of leptin are found in patients with higher the degree of advancement of endometrial cancer and lower levels of histopathological differentiation [22]. A correlation has also been established between leptin concentrations and lymphatic vessel infiltration with lymph node metastases, which is also consistent with our findings above. Gao et al. also have found that endometrial cancer cell lines expressed higher levels of leptin receptor expression in contrast to primary benign endometrial cells [23]. Both leptin receptor (Ob-R) isoforms, long-form (Ob-Rb), and short-form (Ob-Ra) were expressed in six endometrial cancer cell lines with different histopathological differentiation status.

In the above-mentioned publication, Gao et al. have also shown that leptin stimulates cell proliferation and induces activation of signal transducers and transcription 3 activators (STAT3), extracellular signal-controlled kinase (ERK1/2), AKT and cyclooxygenase (COX) -2 in endometrial cancer cells [23]. It appears that this pathway of signal transduction plays a significant role in the carcinogenesis of endometrial cancer.

Leptin as a hormone increases the expression of fibroblastic growth factors, most notably FGF21 and FGF23. Leptin rapidly stimulates STAT3 phosphorylation in osteoblasts, which suggests that leptin acts directly on bone cells and transduces signaling via the JAK-STAT3 pathway to stimulate FGF23 gene expression. The same signaling pathway also increases FGF21 expression. It seems that both leptin and the above-mentioned fibroblast growth factors play a significant role in the carcinogenesis of endometrial cancer by activation of COX-2 dependent on JAK2/STAT3, MAPK/ERK, and PI3K/AKT.

Significantly elevated FGF21 levels were observed in obese patients as compared to patients with normal body weight. The levels of FGF21 were strictly correlated with BMI and WC values. In our study, no correlations could be found between the levels of FGF23 and the bodyweight or BMI values. This can be explained by the roles of FGF21 and FGF23 in the systemic metabolic processes. FGF21 improves glucose tolerance and insulin susceptibility while reducing the plasma and hepatic levels of triglycerides [24]. In addition, we observed a positive correlation between FGF21 and leptin levels. Some of the most recent publications highlight the role of adiponectin and leptin as potential FGF21 mediators [25,26]. Expression of FGF21 is controlled by a stress-activated transcription factor (STAT3) which is also an activator of leptin transcription. In addition, FGF21 is modulated by the key metabolic transcription factors such as PPARα and PPARγ, which are also induced by leptin [27,28]. Yang et al. demonstrated that FGF21 is a strong metabolic regulator improving the metabolism of glucose and lipids and contributing to the reduction in body weight and, most importantly, adipose tissue [29]. Thus, FGF21 is presented as a promising agent for the treatment of obesity and insulin resistance. In our studies, a positive correlation was also observed between FGF23 and leptin. Contrary results were presented by Wójcik et al., who demonstrated that serum FGF23 levels were markedly lower in obese, insulin-resistant patients as compared to the control group [10]. However, most recent publications by Nakashima et al. confirm the strict correlation between FGF23 and leptin. Studies were conducted in a group of hemodialyzed patients. Although detailed mechanisms responsible for the correlation between leptin and FGF23 are not fully understood, we know that leptin directly impacts the synthesis of FGF23 in bone cells [11,30].

In our study, we observed significantly higher levels of leptin and FGF21, but not of FGF23, in overweight and obese patients with endometrial cancer. We confirmed that leptin levels were higher in patients with endometrial cancer and obesity or diabetes compared to the control group, that is, burdened with these diseases. These results seem to be identical to the results of the research of Luhn et al., who, taking into account the BMI correction and the age of the patients, reported similar results. There are reports saying that the correlation between leptin and endometrial cancer disappears after taking BMI into account [31]. However, taking into account the pathomechanism of leptin, its effect on proliferation and angiogenesis, it appears that the studies of Uchikova et al. confirm the key role of leptin in the pathogenesis of endometrial cancer. In addition, the Daley-Brown study, which demonstrated a relationship between leptin and increased aggressiveness and poor prognosis in patients with endometrial type II, where there is no obesity and diabetes appears to confirm the association of leptin with endometrial cancer regardless of associated diseases. Similarly, studies on FGF21 in patients with type II endometrium and slim patients in older age seem to confirm that FGF acts as an independent factor in endometrial cancer, via FGFR 2 and the PI3K/Akt, mTOR signaling pathways. The doubtful results of our research concern a group of patients with FGI. The patient group was too small, and it is difficult to comment on the reliability of the results.

FGF act through 4 receptors by regulating cell proliferation, their metabolism, and apoptosis. Interactions between factors such as FGF21 or FGF23 and FGFR2 occur upon the mediation of Klotho proteins. Somatic mutations of the fibroblast growth factor receptor 2 (FGFR2) tyrosine kinase gene were observed in 12% of endometrial cancer cases [3]. Further signaling elements include PI3K/Akt, mTOR, and MAPK signaling. Gozgit et al. confirmed that inhibition of FGFR2 and mTOR activity, by combined oral treatment of both AP24534 and ridaforolimus in nude mice, exerted a synergistic antitumor effect in an endometrial cancer xenograft model [32]. Currently, in clinical trials AP24534, which is an oral tyrosine kinase inhibitor, blocks both pathways. The first results appear to be very promising by blocking the proliferation of stem cancer cells [33]. Since other proteins of the FGF family exert their effects by direct interactions with FGF receptors, the potential role of non-hormonal FGFs in the carcinogenesis of prostate or lung cancer can be easily explained [34,35].

FGF21 promotes in vivo and in vitro angiogenesis in extrahepatic tissue by inducing the FGFR1 signal in cooperation with the Klotho beta cofactor. However, the role of FGF21 in proliferation and metastasis of the tumor in extrahepatic tissue is still poorly understood. In our studies, we were able not only to observe higher serum levels of leptin and FGF21 in endometrial cancer patients but also to confirm the correlation of FGF21 levels with cancer stage and grade in these patients. In our results, both FGF21 and leptin meet the criteria for a good diagnostic test. AUC for leptin and FGF21 are 0.79 and 0.81. Similarly, the sensitivity and specificity of the two tested proteins appear to be promising in the diagnosis of endometrial cancer.

As confirmed in numerous studies, the leptin hormone is characterized by positive correlations with the clinical stage of endometrial cancer [21]. We are also aware of reports suggesting an important role of FGF21 in the progression of papillary thyroid carcinoma in obese patients [36]. Animal model evidence is also available, as the lack of FGF21 was found to accelerate the transition from hepatic fibrosis to hepatocellular carcinoma in mice fed with a fat-rich diet. However, even the authors of these reports highlight that understanding the integral physiology of FGF21 is a complex matter [37]. In humans, circulatory levels of FGF21 increase with obesity and reflect the increased accumulation of lipids. The situation is different in rodents, and therefore the antiproliferative role of FGF21 demonstrated in mice cannot be directly translated to humans [38]. In our studies, the serum levels of FGF21 were correlated with the clinical stage of the tumor. Importantly, serum levels of FGF21 were higher not only in patients with advanced endometrial cancer but also in patients with early stages of the disease. As reported by Qian et al., circulating FGF21 levels were also correlated with the increased risk of all stages of colorectal cancer as they play a key role in the enhancement of inflammation and the massive proliferation of cancer cells [39].

FGF23 exerts carcinogenic activity in prostate cancer [31]. High expression of this factor was confirmed both in vivo and in vitro in prostate cancer cell lines. The proliferative effect of FGF23 is exerted both directly and indirectly by the accompanying increase in serum calcium levels.

In our studies, a strong correlation was observed between the FGF23 and leptin hormone levels, while no statistically significant increase in the levels of FGF23 was observed in obese patients with endometrial cancer. In addition, the distribution of serum FGF23 levels as the function of tumor staging and grading failed to meet the criteria of a good diagnostic test. It appears that as of this day, FGF23 cannot be used in the diagnostics or prognostics of endometrial cancer.

According to the Mayo Clinic criteria, lymphadenectomy in patients with endometrial cancer is necessary, except for patients identified as low risk. After analyzing the results of our study, it appears that both leptin and FGF21 will predict the stage of tumor progression well, which correlates with the possibility of performing a smaller range (scope) of surgeries in patients who are often obese and burdened with diseases associated to endometrial cancer.

## 5. Conclusions

Both FGF21 and leptin appear to be useful as a diagnostic, as well as showing a correlation with the degree of clinical and histopathological progress in patients with endometrioid endometrial carcinoma. FGF23 does not exhibit the properties of a good diagnostic test, and its concentration does not correlate with the degree of clinical and histopathological progress.

- Compliance with ethical Standards
- Ethics approval and informed consent:

Resolution number: KB-0012/77/12 of the Bioethics Committee of the Pomeranian University of Medicine in Szczecin of 13 October 2012.

All procedures performed in studies involving human participants were in accordance with the ethical standards of the institutional and/or national research committee and with the 1964 Helsinki declaration and its later amendments or comparable ethical standards.

Written informed consent was provided by the patients as well as the physician. All the patients have signed informed consent for the study, which has been signed and initialed by the doctor on each side. Patients carefully read the information and were able to ask questions.

## Figures and Tables

**Table 1:** Examined protein concentrations in relation to endometrium cancer risk factors.

Risk Factor	Patients (*n*)	Leptin (ng/mL) Median (95%CI)	FGF 21 (pg/mL) Median (95%CI)	FGF-23 (pg/mL) Median (95%CI)
BMI 22–25	48	6.9 (3.6–8.8)	134.2 (116.4–149.7)	56.7 (50.5–68.8)
BMI 25–30	76	18.2 (15.4–22.3)	120.8 (126.1–143.8)	74.5 (63.3–78.8)
BMI > 30	58	25.9 (19.1–26.8)	230.1 (136.8–221.4)	87.7 (69.4–91.1)
DM type 2—yes	105	21.6 (4.9–26.8)	241.5 (155.5–267.1)	83.4 (67.6–88.6)
DM type 2—no	77	17.4 (15.1–23.4)	143.8 (133.7–167.7)	72.8 (69.5–91.3)
WC < 100 cm	67	14.8 (11.3–19.0)	118.8 (121.1–148.8)	62.3 (51.5–69.9)
WC > 100 cm	115	21.8 (14.9–29.8)	187.8 (145.6–206.5)	69.1 (65.8–83.2)
PM	68	15.0 (14.6–22.6)	117.4 (131.8–182.2)	66.2 (65.6–73.4)
M	114	12.0 (13.7–24.8)	111.7 (108.6–144.9)	71.3 (65.6–78.7)
Fasting glucose intolerance, yes	21	13.4 (8.8–19.2)	149.6 (136.9–182.1)	79.7 (64.4–79.9)
Fasting glucose intolerance, no	161	14.6 (12.3–18.8)	141.4 (140.7–170.4)	70.7 (63.681.2)

**Abbreviations:** BMI, body mass index; DM, type 2 diabetes mellitus type 2; WC, waist circumference; PM, premenopausal status; M, menopausal status.

**Table 2:** Distribution of patients into subgroups.

Study Group	Median Age (Years)	*n*
All patients	54.43	182
Premenopausal	42.63	68
Postmenopausal	66.68	114
Endometrial cancer	54.36	98
Endometrial endometrioid adenocarcinoma	55.71	82
Non-endometrial endometrioid carcinoma	68.20	16
FIGO I, II	48.96	69
FIGO III, IV	68.95	29
Grade 1	52.34	32
Grade 2	61.08	41
Grade 3	63.44	25
Lymphonode metastasis, Yes	67.81	33
Lymphonode metastasis, No	54.37	65
Lymph vessel involvement, Yes	61.67	52
Lymph vessel involvement, No	56.93	46
Myometrial infiltration depth	55.69	59
Myometrial infiltration superficial	55.04	29
Benign changes endometrium	53.22	84
Normal endometrium	60.04	51
Endometrial polyp	47.12	33

**Table 3:** Relationship between protein concentrations and metabolic disorders in patients from group A and group B.

Diabetes			
	Group A with diabetes	Group B with diabetes	*p*
*n*	64	41	-
%	65.3%	48.9%	-
median leptin (ng/mL)	24.2	19.9	0.01
medianFGF21 (pg/mL)	254.6	229.2	0.001
**Obesity**			
	Group A with obesity	Group B with obesity	*p*
*n*	36	22	-
%	36.7%	26.2%	-
median leptin (ng/mL)	26.7	22.1	0.03
median FGF21 (pg/mL)	251.6	222.5	0.001
**Fasting Glucose Intolerance**			
	Group A with FGI	Group B with FGI	*p*
*n*	11	10	-
%	11.2%	11.9%	-
median leptin (ng/mL)	16.1	12.2	0.001
median FGF21 (pg/mL)	154.8	147.2	NS

**Table 4:** Concentration distribution of examined proteins in endometrium cancer and control group.

Variable	Group A			Group B			*p*
	Patients (*n*)	Range	Median (95% CI)	Patients (*n*)	Range	Median (95% CI)	
Leptin (ng/mL)	98	(0.11–117)	16.9 (11–46.8)	84	(0.4–44.7)	14.1 (12.6–17.0)	0.0004
FGF21 (pg/mL)	98	(144.8–217.7)	181.8 (169.9–222.1)	84	(121.1–188.6)	152.1 (146.3–168.3)	0.003
FGF23 (pg/mL)	98	(61.3–91.4)	81.3 (77.2–86.3)	84	(53.0–71.4)	70.5 (67.8–73.6)	NS

**Table 5:** Concentration distribution of examined proteins in endometrium cancer and subgroups with endometrial polyps.

Variable	Group A			Group B2			*p*
	Patients (*n*)	Range	Median (95% CI)	Patients (*n*)	Range	Median(95% CI)	
Leptin (ng/mL)	98	(0.11–117)	16.9 (11–46.8)	33	(0.4–38.9)	15.3 (12.7–16.4)	0.01
FGF21 (pg/mL)	98	(144.8–217.7)	181.8 (169.9–222.1)	33	(108.1–161.3)	140.7 (146.3–162.6)	0.004
FGF23 (pg/mL)	98	(61.3–91.4)	81.3 (77.2–86.3)	33	(18.0–73.2)	68.1 (65.9–72.6)	NS

**Table 6:** Sensitivity and specificity of individual proteins.

	Leptin Cut Off 17.2 ng/mL	FGF23 Cut Off 74.7 pg/mL	FGF21 Cut Off 176.5 pg/mL
Sensitivity	82%	60%	86%
Specificity	71%	52%	77%

**Table 7:** Multivariate logistic regression analysis.

	OR	95% CI	*p*
BMI (kg/m^2^)	1.26	0.96–1.41	0.02
WC	0.98	0.79–1.06	0.54
DM type 2	0.76	0.59–0.88	0.58
Age	0.92	0.78–1.02	0.02
FGF21	1.21	0.96–1.31	0.03
FGF23	0.66	0.59–0.79	0.48
Leptin	1.24	1.08–1.30	0.01

## References

[1] Zhang S., Gong T.T., Liu F.H., Jiang Y.T., Sun H., Ma X.X., Zhao Y.H., Wu Q.J. (2019). Global, Regional, and National Burden of Endometrial Cancer, 1990–2017: Results from the Global Burden of Disease Study, 2017. Front. Oncol..

[2] De Pergola G., Silvestris F. (2013). Obesity as a major risk factor for cancer. J. Obes..

[3] Taurin S., Yang C.H., Reyes M., Cho S., Coombs D.M., Jarboe E.A., Werner T.L., Peterson C.M., Janát-Amsbury M.M. (2018). Endometrial cancers harboring mutated fibroblast growth factor receptor 2 protein are successfully treated with a new small tyrosine kinase inhibitor in an orthotopic mouse model. Int. J. Gynecol. Cancer.

[4] Kurosu H., Kuro-o M. (2009). The Klotho gene family as a regulator of endocrine fibroblast growth factors. Mol. Cell. Endocrinol..

[5] Koziczak M., Holbro T., Hynes N.E. (2004). Blocking of FGFR signaling inhibits breast cancer cell proliferation through downregulation of D-type cyclins. Oncogene.

[6] Kang Y.E., Kim J.T., Lim M., Oh C., Liu L., Jung S.N., Won H.R., Lee K., Chang J.W., Yi H.S. (2019). Association between circulating fibroblast growth factor 21 and aggressiveness in thyroid cancer. Cancers.

[7] Arnedos M., Andre F., Soria J.C., Dieci M.V. (2013). Fibroblast growth factor receptor inhibitors as a cancer treatment: From a biologic rationale to medical perspectives. Cancer Discov..

[8] Zhang X., Ibrahimi O.A., Olsen S.K., Umemori H., Mohammadi M., Ornitz D.M. (2006). Receptor Specificity of the Fibroblast Growth Factor Family. J. Biol. Chem..

[9] Beenken A., Mohammadi M. (2009). The FGF family: Biology, pathophysiology and therapy. Nat. Rev. Drug Discov..

[10] Wojcik M., Janus D., Dolezal-Oltarzewska K., Drozdz D., Sztefko K., Starzyk J.B. (2012). The association of FGF23 levels in obese adolescents with insulin sensitivity. J. Pediatr. Endocrinol. Metab..

[11] Nakashima Y., Mima T., Yashiro M., Sonou T., Ohya M., Masumoto A., Yamanaka S., Koreeda D., Tatsuta K., Hanba Y. (2016). Expression and localization of fibroblast growth factor (FGF)23 and Klotho in the spleen: Its physiological and functional implications. Growth Factors.

[12] Li J.R., Chiu K.Y., Ou Y.C., Wang S.S., Chen C.S., Yang C.K., Ho H.C., Cheng C.L., Yang C.R., Chen C.C. (2019). Alteration in serum concentrations of FGF19, FGF21, and FGF23 in patients with urothelial carcinoma. BioFactors.

[13] Kharitonenkov A., Shiyanova T.L., Koester A., Ford A.M., Micanovic R., Galbreath E.J., Sandusky G.E., Hammond L.J., Moyers J.S., Owens R.A. (2005). FGF-21 as a novel metabolic regulator. J. Clin. Investig..

[14] Kaess B.M., Barnes T.A., Stark K., Charchar F.J., Waterworth D., Song K., Wang W.Y., Vollenweider P., Waeber G., Mooser V. (2010). FGF21 signalling pathway and metabolic traits- genetic association analysis. Eur. J. Hum. Genet..

[15] Presta M., Chiodelli P., Giacomini A., Rusnati M., Ronca R. (2017). Fibroblast growth factors (FGFs) in cancer: FGF traps as a new therapeutic approach. Pharmacol. Ther..

[16] Mullen M., Gonzalez-Perez R.R. (2016). Leptin-induced JAK/STAT signaling and cancer growth. Vaccines.

[17] Marchelek-Myśliwiec M., Dziedziejko V., Nowosiad-Magda M., Wiśniewska M., Safranow K., Pawlik A., Domański L., Dołęgowska K., Dołęgowska B., Stępniewska J. (2019). Bone Metabolism Parameters in Hemodialysis Patients With Chronic Kidney Disease and in Patients After Kidney Transplantation. Physiol. Res..

[18] Marchelek-Mysliwiec M., Wisniewska M., Nowosiad-Magda M., Safranow K., Kwiatkowska E., Banach B., Dołegowska B., Dołegowska K., Stepniewska J., Domanski L. (2018). Association between Plasma Concentration of Klotho Protein, Osteocalcin, Leptin, Adiponectin, and Bone Mineral Density in Patients with Chronic Kidney Disease. Horm. Metab. Res..

[19] Chui P.C., Antonellis P.J., Bina H.A., Kharitonenkov A., Flier J.S., Maratos-Flier E. (2010). Obesity is a fibroblast growth factor 21 (FGF21)-resistant state. Diabetes.

[20] Daley-Brown D., Oprea-Ilies G.M., Lee R., Pattillo R., Gonzalez-Perez R.R. (2015). Molecular cues on obesity signals, tumor markers and endometrial cancer HHS Public Access Author manuscript. Horm. Mol. Biol. Clin. Investig..

[21] Cymbaluk-Płoska A., Chudecka-Głaz A., Jagodzińska A., Pius-Sadowska E., Sompolska-Rzechuła A., Machaliński B., Menkiszak J. (2018). Evaluation of biologically active substances promoting the development of or protecting against endometrial cancer. Onco Targets Ther..

[22] Zhang Y., Liu L., Li C., Ai H. (2014). Correlation analysis between the expressions of leptin and its receptor (ObR) and clinicopathology in endometrial cancer. Cancer Biomark..

[23] Gao J., Tian J., Lv Y., Shi F., Kong F., Shi H., Zhao L. (2009). Leptin induces functional activation of cyclooxygenase-2 through JAK2/ STAT3, MAPK/ERK, and PI3K/AKT pathways in human endometrial cancer cells. Cancer Sci..

[24] Lundåsen T., Hunt M.C., Nilsson L.M., Sanyal S., Angelin B., Alexson S.E., Rudling M. (2007). PPARα is a key regulator of hepatic FGF21. Biochem. Biophys. Res. Commun..

[25] Quarta C., Sánchez-Garrido M.A., Tschöp M.H., Clemmensen C. (2016). Renaissance of leptin for obesity therapy. Diabetologia.

[26] Funcke J.B., Scherer P.E. (2019). Beyond adiponectin and leptin: Adipose tissue-derived mediators of inter-organ communication. J. Lipid Res..

[27] Muise E.S., Azzolina B., Kuo D.W., El-Sherbeini M., Tan Y., Yuan X., Mu J., Thompson J.R., Berger J.P., Wong K.K. (2008). Adipose fibroblast growth factor 21 is up-regulated by peroxisome proliferator-activated receptor γ and altered metabolic states. Mol. Pharmacol..

[28] Badman M.K., Pissios P., Kennedy A.R., Koukos G., Flier J.S., Maratos-Flier E. (2007). Hepatic Fibroblast Growth Factor 21 Is Regulated by PPARα and Is a Key Mediator of Hepatic Lipid Metabolism in Ketotic States. Cell Metab..

[29] Yang C., Wang C., Ye M., Jin C., He W., Wang F., McKeehan W.L., Luo Y. (2012). Control of lipid metabolism by adipocyte FGFR1-mediated adipohepatic communication during hepatic stress. Nutr. Metab..

[30] Nakashima A., Yokoyama K., Kawanami D., Ohkido I., Urashima M., Utsunomiya K., Yokoo T. (2018). Association between resistin and fibroblast growth factor 23 in patients with type 2 diabetes mellitus. Sci. Rep..

[31] Gupta A., Herman Y., Ayers C., Beg M.S., Lakoski S.G., Abdullah S.M., Johnson D.H., Neeland I.J. (2016). Plasma leptin levels and risk of incident cancer: Results from the dallas heart study. PLoS ONE.

[32] Gozgit J.M., Squillace R.M., Wongchenko M.J., Miller D., Wardwell S., Mohemmad Q., Narasimhan N.I., Wang F., Clackson T., Rivera V.M. (2013). Combined targeting of FGFR2 and mTOR by ponatinib and ridaforolimus results in synergistic antitumor activity in FGFR2 mutant endometrial cancer models. Cancer Chemother. Pharmacol..

[33] Kim D.H., Kwak Y., Kim N.D., Sim T. (2016). Antitumor effects and molecular mechanisms of ponatinib on endometrial cancer cells harboring activating FGFR2 mutations. Cancer Biol. Ther..

[34] Feng S., Wang J., Zhang Y., Creighton C.J., Ittmann M. (2015). FGF23 promotes prostate cancer progression. Oncotarget.

[35] Dey J.H., Bianchi F., Voshol J., Bonenfant D., Oakeley E.J., Hynes N.E. (2010). Targeting fibroblast growth factor receptors blocks PI3K/AKT signaling, induces apoptosis, and impairs mammary tumor outgrowth and metastasis. Cancer Res..

[36] Bernard R.S., Zheng L., Liu W., Winer D., Asa S.L., Ezzat S. (2005). Fibroblast growth factor receptors as molecular targets in thyroid carcinoma. Endocrinology.

[37] Yang C., Lu W., Lin T., You P., Ye M., Huang Y., Jiang X., Wang C., Wang F., Lee M.H. (2013). Activation of Liver FGF21 in hepatocarcinogenesis and during hepatic stress. BMC Gastroenterol..

[38] Singhal G., Kumar G., Chan S., Ma Y., Vardeh H.G., Nasser I.A., Flier J.S., Maratos-Flier E. (2018). Deficiency of fibroblast growth factor 21 (FGF21) promotes hepatocellular carcinoma (HCC) in mice on a long term obesogenic diet. Mol. Metab..

[39] Qian J., Tikk K., Weigl K., Balavarca Y., Brenner H. (2018). Fibroblast growth factor 21 as a circulating biomarker at various stages of colorectal carcinogenesis. Br. J. Cancer.

